# Assessing the impact of voluntary food reformulation targets: Mid-point assessment of Australia’s voluntary sodium and saturated fat reduction policy

**DOI:** 10.1038/s41430-025-01647-5

**Published:** 2025-07-24

**Authors:** Daisy H. Coyle, Liping Huang, Monica Hu, Nadine Ghammachi, Simone Pettigrew, Jason H. Y. Wu

**Affiliations:** 1 The George Institute for Global Health, University of New South Wales, Level 18, International Towers 3300 Barangaroo Ave, Barangaroo, NSW 2000 Australia; 2 School of Public Health, University of California, Berkeley, CA USA; 3 School of Public Health, UNSW Sydney, Sydney, NSW Australia

**Keywords:** Nutrition, Epidemiology

## Abstract

**Background/objectives:**

Most reformulation initiatives worldwide are implemented through voluntary measures. Despite the reliance on voluntary targets, there is limited evidence of their effectiveness. This study aimed to assess the impact of Australia’s voluntary sodium and saturated fat reformulation policy halfway through its four-year implementation period.

**Subjects/methods:**

The 2019 and 2022 FoodSwitch databases provided data on the nutritional composition of packaged foods sold by major Australian supermarket retailers. For the food categories targeted by the policy, we assessed changes between 2019 and 2022 in (i) the overall proportions of products that met the sodium and saturated fat targets and (ii) changes in the proportion of products meeting the targets across the top 10 leading food manufacturers.

**Results:**

Between 2019 and 2022, there was a small increase in the proportion of products meeting the sodium targets (50.0% in 2019 versus 57.5% in 2022, *p* < 0.001). Across the top 10 manufacturers that sold products subject to a sodium target, seven made progress towards meeting the targets (ranging from +1.6% to +30.2%). For saturated fat, the proportion of products meeting the targets didn’t change (61.1% in 2019 versus 60.2% in 2022, *p* = 0.74) and nine of the 10 top manufacturers did not make any progress towards meeting the targets.

**Conclusion:**

Midway through the implementation period of Australia’s voluntary sodium and saturated fat targets, food manufacturers have made minimal progress towards meeting the targets, especially for saturated fat.

## Introduction

Non-communicable diseases (NCDs) are the leading causes of death worldwide [1]. Diet is one of the main modifiable risk factors in the development of NCDs, with one in every five deaths globally attributable to suboptimal diets [2]. The World Health Organization (WHO) recommends several public health ‘Best buy’ policies for improving diet for the prevention and control of NCDs [3]. Reformulation of packaged food products to eliminate trans fatty acids and reduce levels of sodium, saturated fat and free sugars is one of the recommended interventions [3, 4]. In response, many governments have implemented reformulation targets to encourage food manufacturers to reformulate their packaged food products to meet recommended maximum levels of key nutrients [5, 6]. Reformulation programs, usually in the form of voluntary targets, are particularly common in middle- and high-income countries where processed foods are a major source of energy and nutrients in the diet [7, 8].

In Australia, voluntary reformulation targets were introduced in 2020 as part of the Healthy Food Partnership [9]. Founded in 2015, the Healthy Food Partnership is a collaborative initiative between the Australian government, the public health sector, and the food industry to improve dietary intake across the Australian population [10]. A key strategy of the initiative is to encourage the reformulation of selected packaged foods to improve their nutritional profile [9]. The first set of voluntary reformulation targets (Wave 1), released in 2020, include sodium targets across 27 food categories (Supplementary Table 1) and saturated fat targets across five food categories (Supplementary Table 2) [9]. Wave 2 targets were released in 2021 and include sugar targets for nine food categories and additional sodium targets across five food categories [9]. Both waves of targets have four-year implementation periods. A third wave of targets is also planned, but details have not been released.

Prior research has estimated the potential impact of these reformulation targets on household purchases of sodium from packaged food [11–13]. These modeling studies suggested that full implementation of the sodium targets could drive moderate reductions in sodium intake (−107 mg/d per person) and could prevent almost 2000 incident cases of NCDs per year, primarily cardiovascular disease [12]. However, such health outcomes could only be achieved if manufacturers complied with the targets. The aim of this study was to assess the progress made by food manufacturers towards meeting the Australian sodium and saturated fat targets mid-way through the first-wave implementation period, with a particular focus on the leading food manufacturers.

## Materials and methods

### Nutrition composition data

The sodium (mg/100 g) and saturated fat (g/100 g) content in food and beverage products in 2019 (pre policy implementation) and 2022 (mid-point of Wave 1) were obtained from FoodSwitch databases, which are large Australian nutrition composition databases complied annually [14]. Each year, trained data collectors collect in-store nutritional information from packaged food across five major supermarket retailers (Coles, Woolworths, ALDI, IGA, Harris Farm) in the Sydney metropolitan area. Trained data collectors take photographs of all food and beverage products sold in-store. The captured images are then uploaded to a central management system where food packaging information is extracted from the photographs. Extracted data relevant to this study included the product name, package size (g), brand name, manufacturer name, and nutrient content per 100 g or mL and per serve. The products included in FoodSwitch account for >95% of all packaged food products purchased by Australian households (according to Nielsen IQ data) [8].

### Evaluation of the reformulation program

We first identified individual products available in the 2019 and 2022 FoodSwitch databases that were targeted under the Wave 1 sodium targets. Once all products were identified, we then grouped them according to the Healthy Food Partnership food categories (Supplementary Table 1). Products were considered to meet the targets if sodium content was at or below the target level. The same approach was then applied to assess progress against the saturated fat targets (Supplementary Table 2). Using their unique barcodes, products were also classified as either matched (i.e. the product was present in both the 2019 and 2022 databases) or unmatched (the product was present in either 2019 or 2022 but not both).

### Food manufacturers included in analyses

To assess the progress made by major food manufacturers towards compliance with the sodium and saturated fat targets, we identified the top 10 food manufacturers that sold the highest number of total products in 2019 and 2022 across the Healthy Food Partnership food categories. We assessed how their compliance and ranking compared with the other top 10 manufacturers over this period. Analyses were performed separately for sodium and saturated fat given that the major food manufacturers differed for the two nutrients.

### Statistical analysis

We first ascertained the total number of products overall and in each product category targeted under the first wave of the Healthy Food Partnership reformulation targets and the number and proportion of these products that were compliant with the target levels in 2019 and 2022. We then calculated the overall change in the proportion of products meeting the targets in 2019 versus 2022. To test whether changes in the proportions of overall products meeting the targets over that period were significant, we used generalized estimating equations to estimate prevalence ratios. We also evaluated the proportion of products meeting the targets in 2019 and 2022 among the top 10 manufacturers selling the highest number of packaged foods under the targeted food categories, and compared compliance rankings within each year and over time. All analyses were done using R version 4.2.3 and RStudio 2023.06.0 Build 421. Codes used to generate the result can be shared upon request.

## Results

### Overview of included products

The 27 categories covered by the Healthy Food Partnership sodium targets included a total of 7095 products, with 3329 products covered in 2019 and 3,766 covered in 2022. Among these, 1487 were present in both years (matched products), 1842 were present only in 2019 and 2279 were present only in 2022 (unmatched products). For the five food categories with saturated fat targets, 780 products were identified (358 products in 2019 and 422 products in 2022). Among those, 142 products were present in both 2019 and 2022 (matched products), 216 products were only present in 2019, and 280 products were only present in 2022 (unmatched products).

### Change in the proportion of products meeting sodium targets

In 2019, 50.0% of all assessed products met the sodium targets, which increased by 7.5% to 57.5%, in 2022 (Table 1) (*p* < 0.001). From 2019 to 2022, increased compliance with the sodium targets was evident in 19 out of 27 (70%) food categories (increase per category ranged from 0.6% to 39.4%), whereas a reduction in compliance was observed for eight (30%) categories (decrease per category ranged from −7.0% to − 0.6%). The largest improvements were found for “pesto” (39.4% increase in compliance), “leavened bread” (21.9% increase), and “bacon” (21.4% increase). Conversely, compliance worsened for “extruded and pelleted snacks” (7.0% reduction in compliance), “frankfurts and saveloys” (6.6% reduction), and “Asian-style sauces” (6.5% reduction) (Table 1). Among the matched products, 50.4% met the sodium targets in 2019, increasing to 54.2% in 2022 (Supplementary Table 3). Among the unmatched products, 49.7% of products met the sodium targets in 2019, increasing to 59.6% in 2022 (Supplementary Table 4).

**Table 1:** Change in proportion of products (%) meeting sodium targets, overall and for each targeted food category.

Food category	Sub-category	Number of products assessed		Products meeting the targets (%)		Change in % of products meeting targets
		2019	2022	2019	2022	
***All categories***		***3329***	***3766***	***50.0***	***57.5***	***+7.5***
Bread	Leavened breads	329	460	41.3	63.3	***+***21.9
	Flat bread	154	200	44.8	59.0	***+***14.2
Cheese	Cheddar and cheddar style variety cheese products	95	94	64.2	58.5	−5.7
	Processed cheeses	36	39	55.6	51.3	−4.3
Crumbed & battered proteins	Meat and poultry	121	148	39.7	52.7	***+***13.0
	Seafood	79	111	31.7	35.1	***+***3.5
Gravies and sauces	Gravies and finishing sauces	70	90	35.7	50.0	***+***14.3
	Pesto	24	35	29.2	68.6	***+***39.4
	Asian-style sauces	74	86	29.7	23.3	−6.5
	Other savory sauces	289	302	55.0	58.9	***+***3.9
Pizza	Pizza	95	99	51.6	52.5	***+***1.0
Processed meat	Ham	58	58	37.9	48.3	***+***10.6
	Bacon	65	63	23.1	44.4	***+***21.4
	Processed deli meats	49	37	14.3	10.8	−3.5
	Frankfurts and Saveloys	23	19	43.5	36.8	−6.6
Sausages	Sausages	67	86	22.4	40.7	***+***18.3
Savory biscuits	Plain savory crackers and biscuits	160	151	63.8	68.9	***+***5.1
	Plain corn, rice and other ‘grain-cake’ biscuits	24	16	75.0	68.8	−6.3
	Flavored savory biscuits, crackers and ‘grain-cake’ biscuits	197	273	66.5	65.9	−0.6
Savory pastries	Dry pastries	96	134	60.4	68.7	***+***8.2
	Wet pastries	77	84	45.6	54.8	***+***9.4
Savory snacks	Potato snacks	121	143	38.1	49.6	***+***11.6
	Salt and vinegar snacks	18	20	72.2	75.0	***+***2.8
	Extruded and pelleted snacks	63	85	58.7	51.8	−7.0
	Vegetable, grain and other snacks	160	164	50.0	50.6	***+***0.6
Soups	Soups	377	367	53.9	59.4	***+***5.6
Sweet bakery	Cakes, Muffins and Slices	408	402	61.8	69.2	***+***7.4

**Fig. 1:**
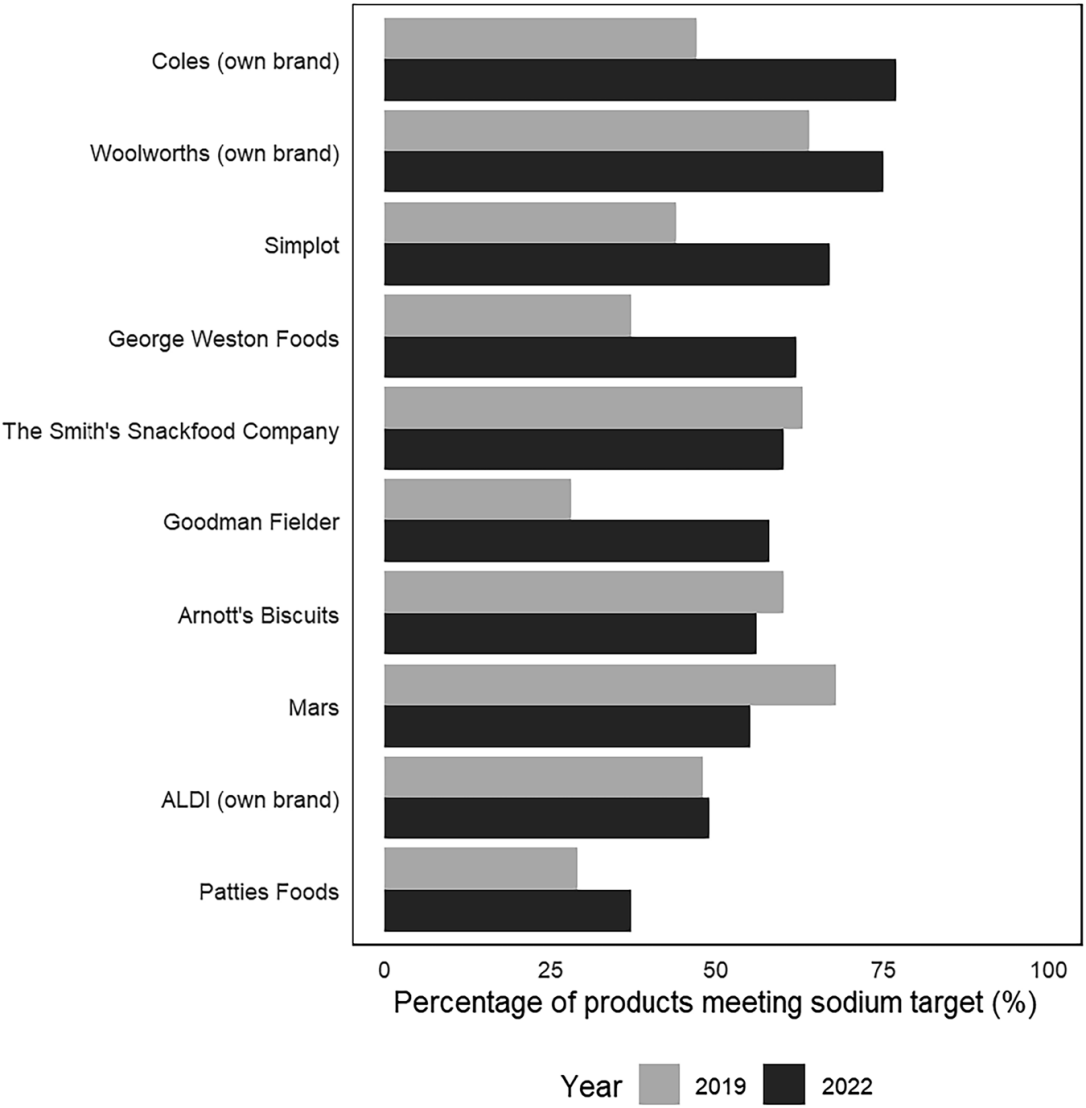
Change in manufacturer ranking over time according to the proportion of products (%) meeting the sodium targets across the top 10 manufacturers selling targeted products. Note: The top 10 food companies are those selling the largest total number of targeted products in 2019 and 2022. Food companies are ranked according to the proportion of their product range that met the sodium targets in 2022.

Between 2019 and 2022, seven of the top 10 companies had an increase in the proportion of products meeting sodium targets (ranging from +1.6% to +30.2%). Three manufacturers (Mars, The Smith’s Snackfood Company, and Arnott’s Biscuits) demonstrated a reduction in compliance (−13.6% to −3.6%) (Fig. 1).

### Change in the proportion of products meeting saturated fat targets

In 2019, 61.1% of products met the saturated fat targets, which decreased to 60.2% in 2022 (Table 2). This change was not statistically significant (p = 0.74). Between 2019 and 2022, four categories had an increase in compliance (ranging from 0.4% to 11.2%) and one category (“Wet pastries”) experienced a reduction in compliance (−17.2%). Among the matched products, 62.0% met the saturated fat targets in 2019, which reduced to 56.3% in 2022 (Supplementary Table 5). Among the unmatched products, 60.7% met the saturated targets in 2019, which increased to 62.1% in 2022 (Supplementary Table 6).

**Table 2:** Change in proportion of products (%) meeting saturated fat targets, overall and for each targeted food category.

Food category	Sub-category	Number of products assessed		Products meeting the targets (%)		Change in % of products meeting targets
		2019	2022	2019	2022	
***All categories***		***358***	***422***	***61.1***	***60.2***	***−1.0***
Pizza	Pizza	95	99	53.7	57.6	+3.9
Processed meat	Frankfurts & Saveloys^*^	23	19	78.3	89.45	+11.2
Sausages	Sausages	67	86	50.8	51.2	+0.4
Savory pastries	Dry pastries	96	134	46.9	54.5	+7.6
	Wet pastries	77	84	92.1	75.0	−17.2

^*^The target for Frankfurts & Saveloys was set as a 10% reduction in products with saturated fat >6.5 g/100 g. For analysis purposes, we set the target level as 6.5 g; products with saturated fat at 6.5 g/100 g or below were considered to meet the target.

**Fig. 2:**
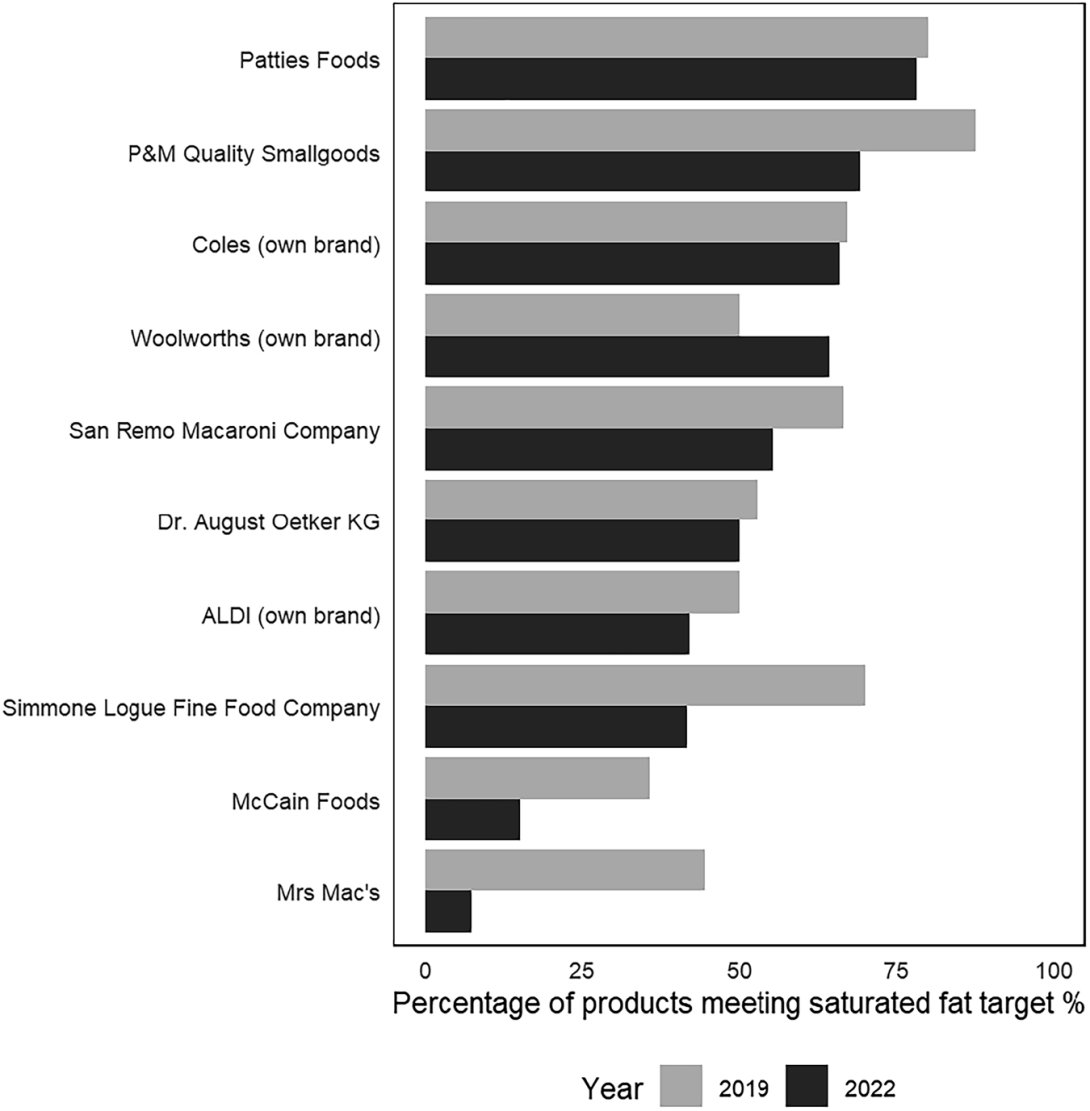
Change in manufacturer ranking over time according to the proportion of products (%) meeting the saturated fat targets across the top 10 manufacturers selling targeted products. Note: The top 10 food companies are those selling the largest total number of targeted products in 2019 and 2022. Food companies are ranked according to the proportion of their product range that met the saturated fat targets in 2022.

Among the top 10 manufacturers selling products with a saturated fat target, only one (Woolworths) had an increase in the proportion of products meeting the saturated fat targets from 2019 to 2022, while the remaining nine had a reduction (ranging from −36.8% to −1.1% in compliance) (Fig. 2).

## Discussion

This mid-point evaluation of Australia’s first wave of reformulation targets under the Healthy Food Partnership found very few achievements have been made. Many leading food companies are not engaging with the program, as evidenced by the minimal progress towards meeting the targets, especially for saturated fat. Since the program’s success relies on food companies reformulating their products, the lack of progress raises serious concerns about the potential of Australia’s reformulation program to achieve meaningful change in the healthiness of the food supply.

Since this evaluation occurred at the mid-point of the first wave of the Healthy Food Partnership reformulation program, the full effect of the targets needs to be reassessed at the end of the implementation period. Nonetheless, the current trajectory of the program is concerning - especially for saturated fat. We observed wide variation in the progress made across the targeted food categories, which suggests food companies are selectively complying with the targets, rather than aiming to reduce sodium and saturated fat levels across all products targeted by the Healthy Food Partnership. This selective compliance highlights a fundamental limitation of relying on voluntary action by the food industry, which is also apparent in the case of other important food policies. For instance, nutrition labeling and declaration of key nutrients have been long recommended by CODEX Alimentarius International Food Standards [15]. However, most food companies only comply once mandatory regulation is enacted [16]. Collectively, our results and those of other voluntary measures attempted in Australia [17–19] suggest that relying on industry commitments will not deliver intended public health outcomes and may instead function as whitewashing exercises for food companies.

While it is positive to see some progress made by the food industry, with seven out of the ten top manufacturers improving their compliance with the sodium targets, especially in some major food categories, our findings indicate that the current rate of progress towards the voluntary targets is unlikely to bring about meaningful reductions in the sodium and saturated fat content of the food supply, even if the effect doubles by the end of the program. While the lack of progress made by food companies is a significant factor, the flawed design of the reformulation program also plays a role. A substantial limitation of the program’s design is the small number of food categories targeted (e.g., the WHO global sodium benchmarks cover almost three times as many food categories) [20]. A second limitation is the weak target levels. When it was designed, the Healthy Food Partnership intended to set reformulation levels at the 33^rd^ percentile for sodium and saturated fat of available foods in the targeted categories to ensure feasibility. However, the present study, in line with previous research [13, 21], has shown that far more than one-third of products *already* met the targets at baseline, suggesting that the targets were more achievable than intended. Our findings suggest that in addition to regulatory measures, there may be a need to redesign the targeted food categories and adjust target levels to ensure they are more ambitious and broader in scope to achieve greater shifts to population diets across Australia.

The Australian Bureau of Statistics (ABS) recently published an evaluation of progress towards meeting the sodium reformulation targets in Australia [22]. Their estimates were based on nutrition composition data from ~1110 packaged food products from Australian companies who voluntarily provided this data along with barcode sales data supplied by major supermarket chains for each of these products [22, 23]. The ABS estimated that progress made towards meeting sodium reformulation targets to date could lead to about 8.3 mg reductions in daily sodium intake, which would represent a ~ 0.4% reduction in daily average sodium intake for Australian adults [22]. Based on the known relationship between sodium intake and blood pressure (and therefore NCD risk), this change will have minimal impact on population health outcomes. This suggests that if the Healthy Food Partnership is to achieve meaningful reductions in disease burden, more stringent and comprehensive targets will be needed [12], such as those covered as part of the WHO global benchmarks [20], as well as incentives for compliance or mandatory regulation to ensure greater compliance with the targets.

The analysis across matched and unmatched products was a novel aspect of this evaluation. We were motivated to conduct this analysis because matched products are those that stay available in the marketplace, and thus tend to be ‘top-sellers’ in any given product category. Reformulation for such products is arguably even more important as they are more commonly consumed. It could be hypothesized that food companies may be reluctant to reformulate top-selling products to avoid changing the taste of the products, which could adversely impact sales. Our findings support this hypothesis, as we found more progress for unmatched products than matched. The limited progress made across matched foods suggests food companies are making minimal effort to reformulate their existing product lines.

Our analysis benefits from the large nutrition databases available from FoodSwitch. The data was collected systematically from products available for sale in large retail stores that collectively account for most of the food products commonly purchased by Australian consumers. Relying on such nationally representative data allows a more accurate and less biased assessment of the progress towards meeting reformulation targets than focusing on subsets of available products or those selectively provided by food manufacturers, such as in the case of the aforementioned ABS evaluation. Another strength was our analysis that explored differences between matched and unmatched products. This allowed us to investigate how much of the progress towards the targets was driven by reformulation versus the formulation of new products and discontinuation of old products lines. A limitation of the study is that we did not explore the impact of the targets on sodium and saturated fat purchases or intakes. Such analyses are needed to explore the impact of the reformulation program on population diets, and should be conducted at the end of the four-year implementation period. This study also only explored the impact of the Wave 1 targets; further research is needed to explore the progress made by the Wave 2 targets that include sodium targets for additional food categories and targets for sugar [24, 25]. Further, future analysis could explore whether there is a difference in locally produced versus imported foods to inform whether overseas based manufacturers are even less likely to comply with the targets.

In conclusion, our analysis of the progress made by the Healthy Food Partnership mid-way through the implementation of the first set of reformulation targets has demonstrated that food companies have made limited progress towards meeting the targets, especially for saturated fat. The fact that many food manufacturers did not reformulate their products highlights the fundamental flaw of the voluntary system. If this trend continues, the reformulation program will not achieve meaningful changes to Australian diets and the health of the population, thereby failing to achieve its stated intentions.

## Supplementary information

Supplementary Materials

## Data Availability

The data that support the findings of this study are available from The George Institute for Global Health. Restrictions apply to the availability of these data, which were used under license for this study. Data are available from the author(s) with the permission of The George Institute for Global Health.
