# Supplementary material for: Assessing the impact of voluntary food reformulation targets: Mid-point assessment of Australia’s voluntary sodium and saturated fat reduction policy
Source: Eur J Clin Nutr. 2025 Jul 24;79(10):1060–5. doi: 10.1038/s41430-025-01647-5 (PMC12537490; doi:10.1038/s41430-025-01647-5)
Supplement: Supplementary file 1 — Supplementary Materials [file 41430_2025_1647_MOESM1_ESM.docx]

# **Supplementary Table 1.** Overview of Wave 1 sodium targets

| **Food category** | **Sub-category** | **Target level (mg/100g)** |
| --- | --- | --- |
| Bread | Leavened bread | 380 |
|  | Flatbread | 450 |
| Cheese | Cheddar and cheddar style variety cheese products | 710 |
|  | Processed cheeses | 1270 |
| Crumbed and Battered Proteins | Meat and poultry | 450 |
|  | Seafood | 270 |
| Gravies and sauces | Gravies and finishing sauces | 450 |
|  | Pesto | 720 |
|  | Asian-style sauces | 680 |
|  | Other savory sauces | 360 |
| Pizza | Pizza | 450 |
| Processed meat | Ham | 1005 |
|  | Bacon | 1005 |
|  | Processed deli meats | 720 |
|  | Frankfurts and Saveloys | 900 |
| Sausages | Sausages | 540 |
| Savory biscuits | Plain Savory crackers and biscuits | 630 |
|  | Plain corn, rice and other ‘grain-cake’ biscuits | 270 |
|  | Flavored savory biscuits, crackers and ‘grain-cake’ biscuits | 720 |
| Savory pastries | Dry pastries | 500 |
|  | Wet pastries | 400 |
| Savory snacks | Potato snacks | 500 |
|  | Salt and vinegar snacks | 810 |
|  | Extruded and pelleted snacks | 720 |
|  | Vegetable, grain and other snacks | 450 |
| Soups | Soups | 280 |
| Sweet Bakery | Sweet Bakery - Cakes, Muffins and Slices | 360 |

# **Supplementary Table 2.** Overview of Wave 1 saturated fat targets **­**

| **Food category** | **Sub-category** | **Target level (g/100g)** |
| --- | --- | --- |
| Pizza | Pizza | 4 |
| Processed meat | Frankfurts & Saveloys | 10% reduction for products with a saturated fat level  >6.5g per 100g |
| Sausages | Sausages | 7 |
| Savory pastries | Dry pastries | 7 |
|  | Wet pastries | 7 |

#

# **Supplementary Table 3.** Change in proportion of products (%) meeting the sodium targets, overall and for each targeted food category among matched products

| **Food Category** | **Sub-category** | **Number of products assessed** | | **Products meeting the targets (%)** | | **Change in % of products meeting targets** |
| --- | --- | --- | --- | --- | --- | --- |
|  |  | **2019** | **2022** | **2019** | **2022** |  |
| ***All categories*** | | ***1,487*** | ***1,487*** | ***50.4*** | ***54.2*** | ***+3.8*** |
| Bread | Leavened breads | 153 | 153 | 43.8 | 54.9 | *+*11.1 |
|  | Flatbread | 76 | 76 | 44.7 | 46.1 | *+*1.3 |
| Cheese | Cheddar and cheddar style variety cheese products | 47 | 47 | 66.0 | 66.0 | 0.00 |
|  | Processed cheeses | 17 | 17 | 47.1 | 47.1 | 0.00 |
| Crumbed & Battered Proteins | Meat and poultry | 44 | 44 | 40.9 | 52.3 | *+*11.4 |
|  | Seafood | 24 | 24 | 45.8 | 41.7 | -4.2 |
| Gravies & sauces | Gravies and finishing sauces | 48 | 48 | 41.8 | 43.8 | *+*2.1 |
|  | Pesto | 12 | 12 | 25.0 | 33.3 | *+*8.3 |
|  | Asian-style sauces | 41 | 41 | 34.2 | 34.2 | 0.00 |
|  | Other savory sauces | 136 | 136 | 52.9 | 55.9 | *+*2.9 |
| Pizza | Pizza | 33 | 33 | 69.7 | 75.8 | *+*6.1 |
| Processed meat | Ham | 26 | 26 | 57.7 | 53.9 | -3.8 |
|  | Bacon | 26 | 26 | 26.9 | 42.3 | *+*15.4 |
|  | Processed deli meats | 17 | 17 | 5.9 | 5.9 | 0.00 |
|  | Frankfurts and Saveloys | 13 | 13 | 38.5 | 46.2 | *+*7.7 |
| Sausages | Sausages | 21 | 21 | 28.6 | 33.3 | *+*4.8 |
| Savory biscuits | Plain savory crackers and biscuits | 84 | 84 | 66.7 | 67.9 | *+*1.2 |
|  | Plain corn, rice and other 'grain-cake' biscuits | 13 | 13 | 69.2 | 69.2 | 0.0 |
|  | Flavored savory biscuits, crackers and 'grain-cake' biscuits | 89 | 89 | 61.8 | 64.0 | *+*2.2 |
| Savory pastries | Dry pastries | 42 | 42 | 52.4 | 59.5 | *+*7.1 |
|  | Wet pastries | 33 | 33 | 36.4 | 36.4 | 0.0 |
| Savory snacks | Potato snacks | 48 | 48 | 37.5 | 50.0 | *+*12.5 |
|  | Salt and vinegar snacks | 14 | 14 | 71.4 | 78.6 | *+*7.1 |
|  | Extruded and pelleted snacks | 22 | 22 | 59.1 | 59. | 0.0 |
|  | Vegetable, grain and other snacks | 68 | 68 | 54.4 | 51.5 | -2.9 |
| Soups | Soups | 181 | 181 | 53.0 | 54.7 | *+*1.7 |
| Sweet Bakery | Cakes, Muffins and Slices | 159 | 159 | 54.1 | 59.1 | *+*5.0 |

# **Supplementary Table 4.** Change in proportion of products (%) meeting the sodium targets, overall and for each targeted food category among unmatched products

| **Food Category** | **Sub-category** | **Number of products assessed** | | **Products meeting the targets (%)** | | **Change in % of products meeting targets** |
| --- | --- | --- | --- | --- | --- | --- |
|  |  | **2019** | **2022** | **2019** | **2022** |  |
| ***All categories*** | | ***1,842*** | ***2,279*** | ***49.7*** | ***59.6*** | ***+9.9*** |
| Bread | Leavened breads | 176 | 307 | 39.2 | 67.4 | +28.2 |
|  | Flatbread | 78 | 124 | 44.9 | 66.9 | +22.1 |
| Cheese | Cheddar and cheddar style variety cheese products | 48 | 47 | 62.5 | 51.1 | -11.4 |
|  | Processed cheeses | 19 | 22 | 63.2 | 54.6 | -8.6 |
| Crumbed & Battered Proteins | Meat and poultry | 77 | 104 | 39.0 | 52.9 | +13.9 |
|  | Seafood | 55 | 87 | 25.5 | 33.3 | +7.9 |
| Gravies & sauces | Gravies and finishing sauces | 22 | 42 | 22.7 | 57.1 | +34.4 |
|  | Pesto | 12 | 23 | 33.3 | 87.0 | +53.6 |
|  | Asian-style sauces | 33 | 45 | 24.2 | 13.3 | -10.9 |
|  | Other savory sauces | 153 | 166 | 56.9 | 61.5 | +4.6 |
| Pizza | Pizza | 62 | 66 | 41.9 | 40.9 | -1.0 |
| Processed meat | Ham | 32 | 32 | 21.9 | 43.8 | +21.9 |
|  | Bacon | 39 | 37 | 20.5 | 46.0 | +25.4 |
|  | Processed deli meats | 32 | 20 | 18.8 | 15.0 | -3.8 |
|  | Frankfurts and Saveloys | 10 | 6 | 50.0 | 16.7 | -33.3 |
| Sausages | Sausages | 46 | 65 | 19.6 | 43.1 | +23.5 |
| Savory biscuits | Plain savory crackers and biscuits | 76 | 67 | 60.5 | 70.2 | +9.6 |
|  | Plain corn, rice and other 'grain-cake' biscuits | 11 | 3 | 81.8 | 66.7 | -15.2 |
|  | Flavored savory biscuits, crackers and 'grain-cake' biscuits | 108 | 184 | 70.4 | 66.9 | -3.5 |
| Savory pastries | Dry pastries | 54 | 92 | 66.7 | 72.8 | +6.2 |
|  | Wet pastries | 44 | 51 | 52.3 | 66.7 | +14.4 |
| Savory snacks | Potato snacks | 73 | 95 | 38.4 | 49.5 | +11.1 |
|  | Salt and vinegar snacks | 4 | 6 | 75.0 | 66.7 | -8.3 |
|  | Extruded and pelleted snacks | 41 | 63 | 58.5 | 49.2 | -9.3 |
|  | Vegetable, grain and other snacks | 92 | 96 | 46.7 | 50.0 | +3.3 |
| Soups | Soups | 196 | 186 | 54.6 | 64.0 | +9.4 |
| Sweet Bakery | Cakes, Muffins and Slices | 249 | 243 | 66.7 | 75.7 | +9.1 |

# **Supplementary Table 5.** Change in proportion of products (%) meeting the saturated fat targets, overall and for each targeted food category among matched products

| **Food category** | **Sub-category** | **Number of products assessed** | | **Products meeting the targets (%)** | | **Change in % of products meeting targets** |
| --- | --- | --- | --- | --- | --- | --- |
|  |  | **2019** | **2022** | **2019** | **2022** |  |
| ***All categories*** | | ***142*** | ***142*** | ***62.0*** | ***56.3*** | ***-5.6*** |
| Pizza | Pizza | 33 | 33 | 42.4 | 33.3 | -9.1 |
| Processed meat | Frankfurts & Saveloys* | 13 | 13 | 84.6 | 84.6 | 0.00 |
| Sausages | Sausages | 21 | 21 | 57.1 | 61.9 | +4.8 |
| Savory pastries | Dry pastries | 42 | 42 | 50.0 | 42.9 | -7.1 |
|  | Wet pastries | 33 | 33 | 90.9 | 81.8 | -9.1 |

*The target for Frankfurts & Saveloys was set as a 10% reduction in products with saturated fat >6.5g. For analysis purposes, we set the target level as 6.5g; products with saturated fat at 6.5g or below were considered to meet the target.

# **Supplementary Table 6.** Change in proportion of products (%) meeting the saturated fat targets, overall and for each targeted food category among unmatched products

| **Food category** | **Sub-category** | **Number of products assessed** | | **Products meeting the targets (%)** | | **Change in % of products meeting targets** |
| --- | --- | --- | --- | --- | --- | --- |
|  |  | **2019** | **2022** | **2019** | **2022** |  |
| ***All categories*** | | ***216*** | ***280*** | ***60.7*** | ***62.1*** | ***+1.5*** |
| Pizza | Pizza | 62 | 66 | 59.7 | 69.7 | +10.0 |
| Processed meat | Frankfurts & Saveloys* | 10 | 6 | 70.0 | 100.0 | +30.0 |
| Sausages | Sausages | 46 | 65 | 47.8 | 47.7 | -0.1 |
| Savory pastries | Dry pastries | 54 | 92 | 44.4 | 59.8 | +15.3 |
|  | Wet pastries | 44 | 51 | 93.2 | 70.6 | -22.6 |

*The target for Frankfurts & Saveloys was set as a 10% reduction in products with saturated fat >6.5g. For analysis purposes, we set the target level as 6.5g; products with saturated fat at 6.5g or below were considered to meet the target.
